# The effects of C2 instability on cervical curvature changes and clinical outcomes after sub-axial cervical expansive door-open laminoplasty

**DOI:** 10.3389/fsurg.2025.1661963

**Published:** 2025-11-03

**Authors:** Liang Ma, Yuntao Liu, Yanhai Xi, Changgui Shi, Xiangyu Meng

**Affiliations:** 1 Department of Minimal Invasive Spine Surgery, The Sixth Affiliated Hospital of Xinjiang Medical University, Urumqi, Xinjiang Uygur Autonomous Region, China; 2 Department of Minimal Invasive Spine Centre, The Second Affiliated Hospital of the Naval Military Medical University, Shanghai, China

**Keywords:** lower cervical vertebra, cervical spondylotic myelopathy, single door enlarged spinal canal plasty, sagittal plane balance, cervical instability

## Abstract

**Objective:**

To investigate the impact of C2 vertebral instability on the sagittal parameters of the cervical spine and the clinical efficacy after cervical laminoplasty with unilateral open-door cervical expansive laminoplasty (EMOL).

**Methods:**

In a retrospective analysis of 18 patients with cervical 2 vertebral instability from August 2017 to August 2021 in the second Affiliated Hospital of Naval Military Medical University and the Six Affiliated Hospital of Xinjiang Medical University treated with single open-door vertebroplasty (C3-6 or C3-7), 36 patients with stable cervical 2 cervical EMOL during the same period (control group). To evaluate the changes in sagittal parameters before and after surgery in the two groups, including C0-2 Cobb angle, C2-7 sagittal axis distance (sagittal vertical axis, SVA), C2-7 Cobb angle, T1 tilt angle (T1-Sl); The postoperative outcome was evaluated using the visual analogue score for neck and shoulder pain (visual analog scale, VAS) and the Japan Society Cervical Function Score (Japanese 0rthopaedic Association, JOA).

**Results:**

Compared to preoperative values, both the observation group and the control group showed significant improvement in postoperative VAS scores and JOA scores. The JOA scores were 14.0 ± 1.6 and 13.1 ± 1.6, with improvement rates of 68.42% and 58.06%, respectively, compared to their respective preoperative scores. However, there was no significant difference between the two groups. The observation group had significantly greater cervical range of motion (ROM) before surgery compared to the control group (*p* < 0.05). At the last follow-up, the observation group showed a significant decrease in C2-7 Cobb angle from preoperative (8.2 ± 2.5)° to (5.1 ± 2.5)° (*p* < 0.05). Cervical ROM decreased from preoperative (39.8 ± 3.6)° to (31.6 ± 4.5)° (*p* < 0.05). C0-2 Cobb angle increased from preoperative (22.0 ± 3.7)° to (25.8 ± 3.1)° (*p* < 0.05). C2-7 SVA increased from preoperative (−19.6 ± 3.4)° to (−15.8 ± 3.7)° (*p* < 0.05). However, there was no significant change in T1 slope at the last follow-up (*p* > 0.05). The observation group showed a decrease in C2 vertebral displacement from preoperative (4.5 ± 0.9) mm to (3.3 ± 0.5) mm (*p* < 0.05), while the C2/3 angle showed no significant change compared to preoperative values (*p* > 0.05). In both groups, postoperative follow-up showed a significant increase in C0-2 Cobb angle and C2-7 SVA, a non-significant difference in T1 slope, and a significant decrease in C2-7 Cobb angle and cervical ROM compared to preoperative values. However, there were no significant differences between the two groups in the above-mentioned parameters (*p* > 0.05).

**Conclusion:**

C2 vertebral instability does not affect the sagittal parameters and efficacy of cervical laminoplasty with EMOL. EMOL surgery for cervical myelopathy with C2 vertebral instability is effective and reliable, without exacerbating C2 vertebral instability. Furthermore, it maintains good sagittal balance of the cervical spine.

## 1 Introduction

Multisegmental cervical spondylotic myelopathy (MCSM), cervical spinal stenosis, and ossification of the posterior longitudinal ligament (OPLL) are pathological changes that cause compression of the cervical spinal cord (1). Patients often present with sensory and motor deficits in the limbs, as well as urinary and fecal dysfunction. With changes in lifestyle, the incidence of these conditions has increased, significantly impacting patients' quality of life and being a major cause of disability. Posterior cervical expansive door-open laminoplasty (EODL) is a primary surgical approach for treating multisegmental cervical spinal cord compression (2, 3). EODL has been proven to have clear and long-lasting efficacy in improving patients’ neurological function and relieving cervical spinal cord compression (4). EODL is a non-fusion surgical technique that preserves cervical spine mobility to a certain extent. Moreover, EODL does not increase cervical instability in patients with pre-existing instability (5). Additionally, EODL preserves the posterior column structures, effectively reducing surgical trauma and postoperative complications (6). However, due to the extensive dissection of muscles, ligaments, and other structures during the procedure, there is a possibility of changes in sagittal parameters of the cervical spine, which may affect clinical outcomes or lead to postoperative axial pain (7, 8). The C2 vertebra plays a crucial role in measuring sagittal parameters of the cervical spine, as multiple parameters are measured with reference to the C2 vertebra. However, there is limited research on whether C2 vertebral instability affects the sagittal parameters and clinical outcomes of lower cervical spine EODL (9). This study aims to retrospectively analyze the clinical and radiographic data of patients who underwent lower cervical spine EODL in our orthopedic department to evaluate the impact of C2 vertebral instability on sagittal parameters and clinical outcomes after EODL.

## 2 Subjects and methods

### 2.1 General information

In this study, the clinical data and follow-up records of patients undergoing cervical EODL in the second Affiliated Hospital of Naval Military Medical University and the Six Affiliated Hospital of Xinjiang Medical University from August 2017 to August 2021 were selected. The patients were divided into the cervical 2 vertebral instability group (observation group) and the cervical 2 vertebral stabilization group (control group), and the patients treated by EODL surgical treatment in the same time according to the age, sex, body mass index, disease duration, surgical segment, follow-up time, operation time, bleeding volume, and complications matching (1:2). All patients signed the informed consent before surgery, and the study was approved by the hospital ethics committee.

### 2.2 Inclusion criteria

(i) Presence of symptoms and signs of cervical spinal cord compression before surgery. (ii) Imaging showing evidence of Multisegmental Cervical Spondylotic Myelopathy (MCSM) or Ossification of Posterior Longitudinal Ligament (OPLL). (iii) Underwent lower cervical spine EODL surgery. (iiii) Follow-up for at least 2 years with complete imaging data.

### 2.3 Exclusion criteria

(i) Presence of cervical radiculopathy and concurrent radicular cervical spine disease. (ii) Concurrent posterior cervical pedicle screw-rod fixation or anterior cervical plate and screw fixation. (iii) History of previous cervical spine surgery or trauma. (iiii) Severe cervical kyphosis deformity or presence of cervical spine infection, tumor, etc. (iiiii) Concurrent rheumatoid arthritis or ankylosing spondylitis, and other related diseases.

According to the inclusion and exclusion criteria, a total of 18 patients were selected for the observation group (C2 vertebral body instability group: x-rays show an angle of more than 11°between the vertebral bodies, or the horizontal displacement between the vertebral bodies exceeds 3.5 m m^10^), including 12 males and 6 females. The age ranged from 45 to 72 years, with an average age of (58.2 ± 3.1) years. The body mass index (BMI) ranged from 18 to 27 kg/m^2^, with an average BMI of (23.8 ± 2.3) kg/m^2^. The duration of the disease ranged from 5 to 14 months, with an average duration of (9.7 ± 5.5) months. Two patients had concomitant hypertension, and one patient had diabetes. Among the patients, 8 underwent surgery at the C3-6 level, and 10 underwent surgery at the C3-7 level. The control group consisted of 36 matched patients, including 25 males and 11 females. The age ranged from 45 to 72 years, with an average age of (58.5 ± 2.9) years. The body mass index (BMI) ranged from 17 to 28 kg/m^2^, with an average BMI of (23.5 ± 2.1) kg/m^2^. The duration of the disease ranged from 6 to 13 months, with an average duration of (9.5 ± 5.6) months. Three patients had concomitant hypertension, and two patients had diabetes. Among the patients, 26 underwent surgery at the C3-6 level, and 10 underwent surgery at the C3-7 level.

### 2.4 Surgical method

The patient is placed under general anesthesia and positioned prone on a plaster bed. The surgical field is prepared with routine disinfection and draping. A midline incision is made posterior to the neck, extending from the spinous processes of C2 to C7, with a length of 8–10 cm. The skin, subcutaneous tissue, fascia, and nuchal ligament are dissected using an electric scalpel. The paraspinal muscles are then dissected bilaterally along the periosteum, exposing the area from the lower half of the C2 spinous process to the spinous processes and laminae of C3-6 or C3-7. Using a burr, slots are created at the junction of the lamina and lateral mass on the side with more compression, which serves as the “open-door” side. The laminae are thinned and removed bilaterally, deep to the spinal canal. On the side with less compression, known as the “hinge” side, the laminae are thinned and the ventral cortex is preserved. The laminae of C3-6 or C3-7 are sequentially lifted from caudal to cranial, and mini titanium plates (arch plates) are used to fixate the lateral mass and laminae on the open-door side. Any remaining bone in the inner edge of the lateral mass and intervertebral foramen area is carefully removed using rongeurs and curettes. Adhesions between the dura mater or nerve roots and the ligamentum flavum are released. Hemostasis is ensured throughout the procedure. After confirming satisfactory pulsation of the dura mater, the surgical site is irrigated with saline solution, and drainage is placed before closing the incision layer by layer. Postoperatively, the patient is instructed to wear a cervical collar for 2–3 weeks as per routine.

### 2.5 Observed indicators

#### 2.5.1 Measurement of sagittal parameters

**Figure 1:**
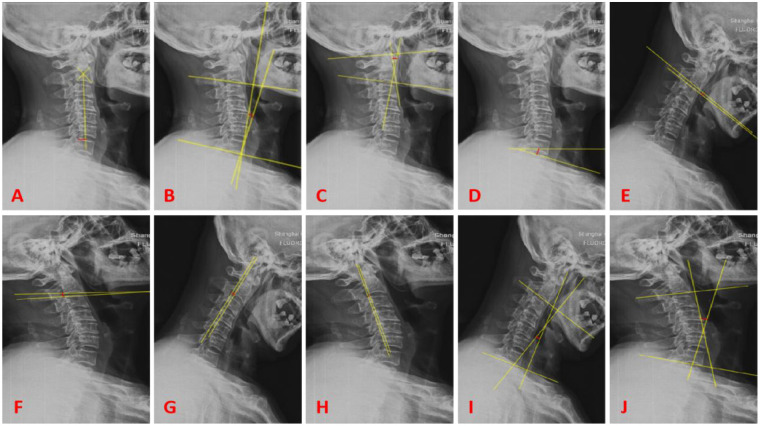
**(A)** Represents C2-7 SVA (red line); **(B)** represents C2-7 cobb angle (red line); **(C)** represents C0-2 cobb angle (red line); **(D)** represents T1-slope (red line); **(E,F)** represent the difference in angle (red line) for the motion angle of C2/3 gap; **(G,H)** represent the difference (red line) for the displacement of C2 vertebral body; **(I,J)** represent the difference (red line) for ROM.

Preoperative and postoperative cervical spine lateral, flexion-extension, CT, and MRI images are obtained. The PACS 3.0 system software is used to measure the following sagittal parameters on cervical x-ray images: C0-2 Cobb angle, C2-7 sagittal vertical axis (SVA) in millimeters, C2-7 Cobb angle, T1-Slope, range of motion (ROM) of the cervical spine, displacement of the C2 vertebral body (measured as the distance between the posterior edge of the C2 vertebral body and the posterior edge of the C3 vertebral body on flexion-extension x-ray images in millimeters), and the angle of motion at the C2/3 level (measured as the difference in angle at the C2/3 level on flexion-extension x-ray images). Two physicians independently measure the imaging parameters twice using the described method. The average value of the measurements is taken as the final measurement data. The assessors are blinded to the study content. Please refer to Figure 1 for specific measurement methods.

#### 2.5.2 Clinical efficacy assessment

Preoperative and postoperative pain and neurological function improvement in patients are evaluated using the visual analog scale (VAS) for neck and shoulder pain and the Japanese Orthopaedic Association (JOA) score for cervical spine function. The improvement rate (%) is calculated as follows: Improvement rate (%) = (Postoperative JOA score - Preoperative JOA score)/(17 - Preoperative JOA score) × 100%.

#### 2.5.3 Complications

Intraoperative and postoperative complications are recorded, including dural tear, nerve injury, postoperative C5 nerve root palsy, postoperative infection and hematoma, postoperative axial pain, failure of internal fixation, and re-closure.

### 2.6 Statistical methods

The data were analyzed using SPSS 26.0 software. Continuous variables are presented as mean ± standard deviation. Paired sample *t*-tests were used for within-group comparisons of preoperative and postoperative data, while independent sample *t*-tests were used for between-group comparisons. Categorical data were analyzed using chi-square tests or Fisher's exact tests. The correlation between clinical efficacy and changes in imaging parameters was analyzed using Pearson correlation tests. A *p*-value <0.05 was considered statistically significant.

## 3 Results

### 3.1 Experimental flow chart

**Figure 2:**
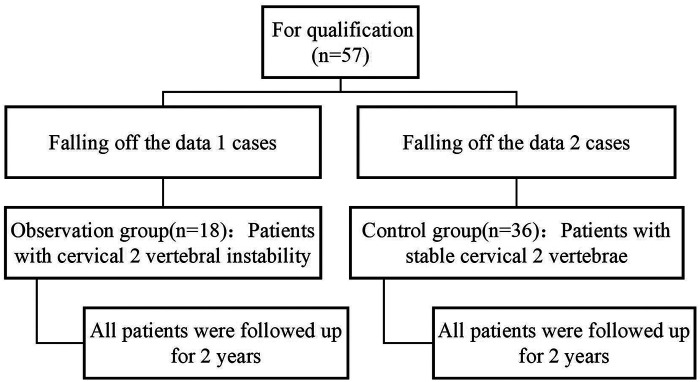
Flow chart of test grouping.

The flow chart of the two groups is shown in Figure 2.

### 3.2 Comparison of preoperative data between the two groups

Baseline data on age, sex, body mass index, disease duration, surgical segment, follow-up time, operation time, bleeding volume, and complications were comparable (*p* > 0.05). See Table 1.

**Table 1:** Basic preoperative data of the two groups.

Items	Observation group (*n* = 18)	Control group (*n* = 36)	χ^2^/Fisher's exact test	*P*
Age	58.2 ± 3.1	58.5 ± 2.9	0.587	0.561
Sex (*n*)	12/6	25/11	0.468	0.494
Disease course (month)	9.7 ± 5.5	9.5 ± 5.6	1.563	0.134
Follow-up time (years)	2.4 ± 0.4	2.9 ± 1.3	1.509	0.141
BMI (kg/m^2^)	23.8 ± 2.3	23.5 ± 2.1	1.489	0.152
Diagnosis (*n*)				
OPLL disease	6/33.3	10/27.8	0.139	0.933
Cervical spinal stenosis	10/56.6	22/61.1		
Cervical herniated disc	2/11.1	4/11.1		
Surgical segment (*n*)				
C3-6	8/44.4	26/72.2	0.643	0.423
C3-7	10/55.6	10/27.8		
Operation time (min)	112.2 ± 3.8	114.2 ± 3.7	1.595	0.120
Bleeding volume (ml)	95.0 ± 6.8	97.5 ± 4.6	1.293	0.205
Complication (*n*)				
Axial symptoms	3/16.7	2/5.6	0.232	0.630
Wound infection	0/0.0	1/2.8	0.509	0.475
C5 nerve root paralysis	1/5.6	3/8.3	0.135	0.713

### 3.3 Neurological function assessment and vas scores

During the postoperative follow-up, it was observed that the JOA score in the observation group improved from 7.5 ± 1.2 preoperatively to 14.0 ± 1.6 postoperatively, with an improvement rate of 68.42%. In the control group, the JOA score improved from 7.7 ± 1.0 preoperatively to 13.1 ± 1.6 postoperatively, with an improvement rate of 58.06%. There were no significant differences in JOA scores between the two groups at the last follow-up. It was also noted that the VAS scores for neck pain significantly decreased in both groups during the postoperative follow-up. See Table 2.

**Table 2:** Neurological function assessment and vas scores.

Items	Observation group (*n* = 18)	Control group (*n* = 36)	*t*	*P*
Neck VAS score				
Preoperative	7.3 ± 0.5	7.3 ± 0.9	0.024	0.981
The last follow-up	1.1 ± 0.1	1.0 ± 0.2	0.711	0.482
JOA score				
Preoperative	7.5 ± 1.2	7.7 ± 1.0	0.562	0.578
The last follow-up	14.0 ± 1.6	13.1 ± 1.6	1.870	0.067

### 3.4 Evaluation of radiographic parameters

**Figure 3:**
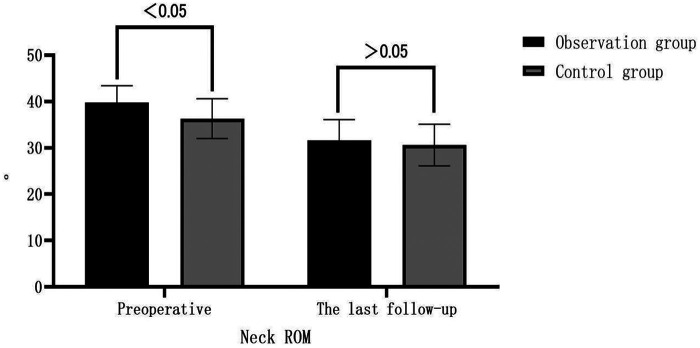
Comparison of patient neck ROM in the two treatment groups.

Before the surgery, the cervical ROM was (39.8 ± 3.6)° in the observation group and (36.3 ± 4.3)° in the control group, with a statistically significant difference (*P* < 0.05). At the last follow-up in the observation group, compared to preoperative values, the C0-2 Cobb angle increased from (22.0 ± 3.7)° to (25.8 ± 3.1)°; C2-7 SVA increased from (−19.6 ± 3.4) mm to (−15.8 ± 3.7) mm; C2-7 Cobb angle decreased from (8.2 ± 2.5)° to (5.1 ± 2.5)°; and cervical ROM decreased from (39.8 ± 3.6)° to (31.6 ± 4.5)°. All these parameters showed statistically significant differences within the observation group (*P* < 0.05), while there was no significant difference in T1-Slope (*P* > 0.05). In the control group, at the last follow-up compared to preoperative values, the C0-2 Cobb angle increased from (22.8 ± 2.7)° to (25.3 ± 2.9)°; C2-7 SVA increased from (−18.7 ± 3.5) mm to (−15.3 ± 1.4) mm; C2-7 Cobb angle decreased from (8.2 ± 2.7)° to (4.9 ± 0.8)°; and cervical ROM decreased from (36.3 ± 4.3)° to (30.6 ± 4.5)°. All these parameters showed statistically significant differences within the control group (*P* < 0.05), while there was no significant difference in T1-Slope (*P* > 0.05). There were no significant differences between the two groups in the above-mentioned parameters at the last follow-up compared to preoperative values (*P* > 0.05). See Table 3. In the observation group, at the last follow-up compared to preoperative values, the displacement of the C2 vertebral body decreased from (4.5 ± 0.9) mm to (3.3 ± 0.5) mm, which was statistically significant (*P* < 0.05), see Figure 3, while the angle of motion at the C2/3 level decreased from (9.9 ± 1.7)° to (8.6 ± 2.5)°, but the difference was not statistically significant (*P* > 0.05). See Table 4.

**Table 3:** Sagittal parameters of the cervical spine in the Two groups of patients.

Items	Observation group (*n* = 18)	Control group (*n* = 36)	*t*	*P*
C_0−2_ Cobb (°)				
Preoperative	22.0 ± 3.7	22.8 ± 2.7	0.716	0.479
The last follow-up	25.8 ± 3.1	25.3 ± 2.9	1.523	0.137
C_2−7_ SVA (mm)				
Preoperative	−19.6 ± 3.4	−18.7 ± 3.5	0.898	0.376
The last follow-up	−15.8 ± 3.7	−15.3 ± 1.4	0.655	0.517
C_2−7_ Cobb (°)				
Preoperative	8.2 ± 2.5	8.2 ± 2.7	0.000	1.000
The last follow-up	5.1 ± 2.5	4.9 ± 0.8	0.440	0.687
T1-Slope (°)				
Preoperative	27.0 ± 5.0	26.1 ± 3.4	0.616	0.542
The last follow-up	26.4 ± 2.8	26.0 ± 2.5	0.464	0.646
Neck ROM (°)				
Preoperative	39.8 ± 3.6	36.3 ± 4.3	4.454	0.013
The last follow-up	31.6 ± 4.5	30.6 ± 4.5	0.666	0.510

**Table 4:** Assessment of the degree of C2 instability in the observation group before surgery and at the last follow-up visit.

Items	Preoperative	Postoperative follow-up	*t*	*P*
C2 vertebral displacement value (mm)	4.5 ± 0.9	3.3 ± 0.5	4.163	0.020
C 2/3 gap activity angle (°)	9.9 ± 1.7	8.6 ± 2.5	0.926	0.351

### 3.5 Complications

In the observation group, 3 cases (16.67%) experienced postoperative axial pain, while in the control group, there were 2 cases (5.56%). One case in the observation group developed C5 nerve root palsy, while in the control group, 3 cases developed C5 nerve root palsy. All patients in both groups achieved recovery through conservative treatment. One case in the control group experienced postoperative wound infection (2.8%), while no infection cases were observed in the observation group. Neither group experienced cerebrospinal fluid leakage, nerve injury, failure of internal fixation, re-closure, or hinge fracture. See Table 5.

**Table 5:** Relationship between changes in sagittal parameters and clinical efficacy in the observation group.

Items		Neck 2 vertebral displacement value (mm)	C2-7 Cobb (°)	Neck ROM (°)
Neck VAS score	r	0.199	0.021	0.003
	*P*	0.428	0.901	0.986
Rate of neurological improvement (%)	r	0.327	0.128	0.022
	*P*	0.185	0.612	0.929

Cervical ROM is cervical mobility; neurological improvement rate: (postoperative JOA score-preoperative JOA score)/(17-preoperative JOA score) 100%.

#### 3.5.1 Relationship between changes in sagittal parameters and clinical efficacy in the observation group

The relationship between changes in sagittal parameters and clinical efficacy in the observation group is shown in Table 5. The improvement rate of neurological function was 68.42%. However, there was no significant correlation between changes in sagittal parameters and cervical range of motion (ROM) with clinical efficacy (*P* > 0.05).

### 3.6 Typical cases

**Figure 4:**
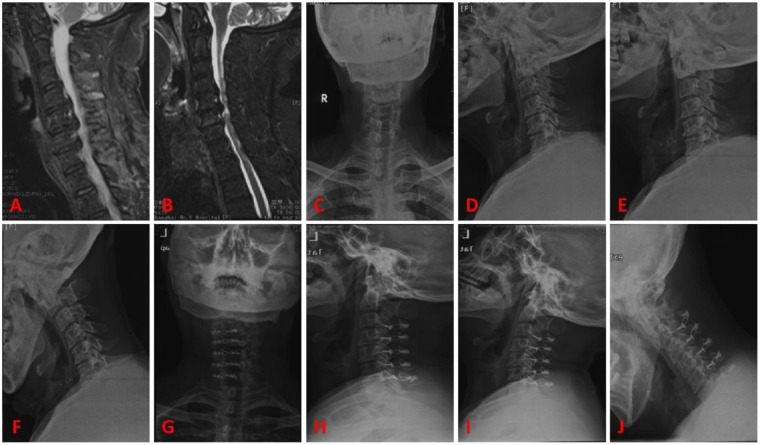
Typical case 1. **(A,B)** Preoperative sagittal MRI images, showing varying degrees of disc protrusion at the C4/5, C5/6, and C6/7 levels. **(C,D)** Preoperative anterior-posterior x-rays, showing loss of cervical lordosis. **(E,F)** Preoperative flexion-extension x-rays, showing instability of the C2 vertebra. **(G,H)** Postoperative follow-up lateral x-rays, showing good position of the internal fixation. **(I,J)** Postoperative follow-up flexion-extension x-rays, showing no significant changes in the instability of the C2 vertebra.

Case 1, female patient, 50 years old. A, B are preoperative sagittal MRI images, showing varying degrees of disc protrusion at the C4/5, C5/6, and C6/7 levels. C, D are preoperative anterior-posterior x-rays, showing loss of cervical lordosis. E, F are preoperative flexion-extension x-rays, showing instability of the C2 vertebra. G, H are postoperative follow-up lateral x-rays, showing good position of the internal fixation. I, J are postoperative follow-up flexion-extension x-rays, showing no significant changes in the instability of the C2 vertebra. See Figure 4.

**Figure 5:**
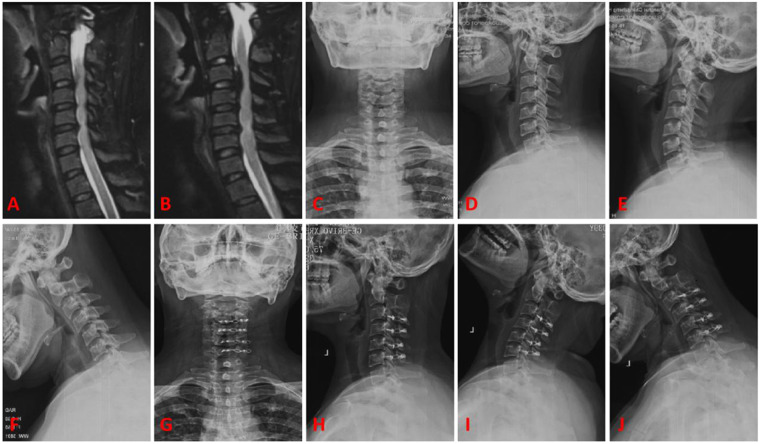
Typical case 2. **(A,B)** Preoperative sagittal MRI images, showing varying degrees of stenosis at the C3/4, C4/5, and C5/6 levels, with ossification of the posterior longitudinal ligament at C3/4. **(C,D)** Preoperative anterior-posterior x-rays, showing slight straightening of the cervical curvature. **(E,F)** Preoperative flexion-extension x-rays, showing instability of the C2 vertebra. **(G,H)** Postoperative follow-up lateral x-rays, showing good position of the internal fixation. **(I,J)** Postoperative follow-up flexion-extension x-rays, showing no significant changes in the instability of the C2 vertebra.

Case 2, male patient, 57 years old. A, B are preoperative sagittal MRI images, showing varying degrees of stenosis at the C3/4, C4/5, and C5/6 levels, with ossification of the posterior longitudinal ligament at C3/4. C, D are preoperative anterior-posterior x-rays, showing slight straightening of the cervical curvature. E, F are preoperative flexion-extension x-rays, showing instability of the C2 vertebra. G, H are postoperative follow-up lateral x-rays, showing good position of the internal fixation. I, J are postoperative follow-up flexion-extension x-rays, showing no significant changes in the instability of the C2 vertebra. See Figure 5.

## 4 Discussion

In the 1970s, Hirabayashi et al. first reported the posterior cervical laminoplasty, which is now widely used for the treatment of MCSM, cervical spinal stenosis, and OPLL (10, 11). It has been proven to be a safe and effective surgical technique (12). However, posterior cervical surgery inevitably causes damage to the muscles and ligaments in the posterior neck region, leading to changes in the normal cervical curvature, disruption of sagittal balance, and even the development of cervical kyphosis (13, 14). Postoperative cervical kyphosis has been observed in 6% to 46% of patients undergoing conventional laminoplasty procedures. In a study by Yang et al., they measured nine sagittal parameters in 164 patients who underwent cervical laminoplasty (15, 16). They found that the lower cervical curvature became straighter (decreased C2-7 Cobb angle) and the center of gravity of the head and neck shifted forward (increased C2-7 SVA). However, compensatory changes were observed in the upper cervical spine and cervicothoracic junction, such as increased cervical overextension (increased C0-2 Cobb angle, T1-Slope) to compensate for these changes (17). Douglas et al. mentioned in their review that for patients with a significant increase in C2-7 SVA or head center of gravity SVA, alternative surgical techniques may be considered as a substitute for laminoplasty, even if there is spinal kyphosis (18). During the C3 laminoplasty procedure, it is usually necessary to dissect the C2 lamina and the distal muscle tissue of the spinous process (19). The posterior bony structures of C2 serve as attachment points for multiple muscles in the posterior head and neck region (20). Excessive dissection and postoperative sagittal imbalance and axial pain may be related to this. The position of the C2 vertebra is of great significance in assessing sagittal balance of the cervical spine. However, most studies on cervical sagittal balance are based on the assumption of C2 vertebral stability. In our study, we focused on the changes in cervical curvature and their clinical significance after EODL surgery, specifically considering the premise of C2 vertebral instability (21).

The results of this study showed that at the last follow-up, both the observation group and the control group showed significant improvement in JOA scores (*P* < 0.05), which is consistent with previous reports on the efficacy of EODL surgery. Furthermore, there was no significant difference in JOA improvement between the observation group and the control group, suggesting that the increased spinal canal space and improved spinal cord function after cervical laminoplasty are not affected by C2 vertebral instability (22–24). Our study found that the degree of C2 vertebral instability did not worsen after surgery in the observation group, and in fact, there was a reduction in C2 vertebral displacement compared to preoperative values (*P* < 0.05). This suggests that laminoplasty not only does not increase C2 vertebral instability but also tends to stabilize the overall cervical spine. This may be attributed to factors such as muscle scarring, ossification of the C2/3 joint capsule, and fusion of inflamed joint surfaces after surgery (25, 26). However, there was a decrease in cervical range of motion compared to preoperative values (*P* < 0.05), indicating that although EODL surgery with C2 vertebral instability leads to a decrease in lower cervical range of motion, it does not affect the stability of the C2 vertebra. Regarding cervical sagittal balance, there were no significant differences in C0-2 Cobb angle, C2-7 SVA, C2-7 Cobb angle, and T1-Slope between the two groups preoperatively. This may be because most patients in the observation group did not show obvious subluxation of the C2 vertebra in the lateral view, but instability was only evident in flexion-extension views (27–29). Therefore, there were no significant differences in these parameters between the two groups in the preoperative lateral x-rays. However, the preoperative range of motion (ROM) of the cervical spine was greater in the observation group compared to the control group, and there was a significant difference (*P* < 0.05). This is related to the increased mobility caused by C2 vertebral instability in the observation group. In both groups, postoperative follow-up showed a significant increase in C0-2 Cobb angle and C2-7 SVA, no significant difference in T1-Slope, and a significant decrease in C2-7 Cobb angle and cervical ROM (30, 31). This indicates an overall forward inclination of the cervical spine. However, there were no significant differences between the groups in the above-mentioned parameters (*P* > 0.05). This is consistent with previous literature reporting that EODL surgery can lead to a straightening of the cervical curvature and even the development of significant kyphotic deformity due to the extensive disruption of the posterior cervical muscles. Cervical kyphosis is also a risk factor for poor postoperative outcomes in patients with cervical myelopathy (32, 33).

The correlation analysis between the changes in these imaging parameters and the clinical efficacy evaluation of the patients shows that the decrease in cervical curvature, reduction in C2 vertebral displacement, and decrease in cervical range of motion are not significantly correlated with the improvement in neck pain and neurological function in patients (34). It can be observed that the postoperative efficacy in patients with MCSM is mainly related to thorough decompression of the spinal cord, rather than being significantly correlated with preoperative C2 vertebral instability (35).

The present study has several limitations that should be acknowledged: (1) Limited sample size: This was a retrospective study, and cases of C2 instability meeting the strict inclusion criteria are relatively uncommon in clinical practice. Although data from two medical centers were included, the sample size of the observation group (*n* = 18) remains small, which may reduce statistical power and increase the risk of Type II error (i.e., failing to detect actual differences that exist). (2) Inherent biases of retrospective design: As a retrospective study, it carries inherent limitations in data collection and analysis, such as selection bias. Future large-scale, multicenter prospective studies are needed to further validate our findings. (3) Unassessed potential influencing factors: Cervical sagittal balance is a complex system that can be influenced by various factors such as pelvic parameters, overall spino-pelvic alignment, and individual muscular compensatory mechanisms. This study did not analyze these more global parameters; future research should take them into consideration.

This study demonstrates that patients with C2 instability underwent significant and specific changes in cervical sagittal parameters after undergoing Expansive Open-Door Laminoplasty (EMOL), including increased global kyphosis (decreased C2–7 Cobb angle), compensatory increase in upper cervical lordosis (increased C0–2 Cobb angle), forward shift in sagittal vertical alignment (increased C2–7 SVA), decreased cervical range of motion (ROM), and reduced C2 vertebral displacement. These changes were not prominent or followed a different pattern in patients with stable C2. Crucially, however, these significant radiological changes did not translate into statistically significant differences in clinical efficacy (improvement in VAS and JOA scores) between the two groups. Therefore, we conclude that although C2 instability leads to specific alterations in postoperative sagittal alignment, it does not negatively impact the final clinical outcomes. EMOL remains an effective and reliable surgical option for patients with cervical spondylotic myelopathy accompanied by C2 instability, as it maintains satisfactory sagittal balance without exacerbating C2 instability.

## Data Availability

The original contributions presented in the study are included in the article/Supplementary Material, further inquiries can be directed to the corresponding authors.
